# Down-regulation of miR-543 expression increases the sensitivity of colorectal cancer cells to 5-Fluorouracil through the PTEN/PI3K/AKT pathway

**DOI:** 10.1042/BSR20190249

**Published:** 2019-03-22

**Authors:** Gang Liu, JianPing Zhou, Ming Dong

**Affiliations:** Department of Gastrointestinal Surgery & Hernia and Abdominal Wall Surgery, The First Hospital, China Medical University, Shenyang 110001, Liaoning, China

**Keywords:** colorectal cancer, chemoresistance, MicroRNA-543, PTEN, 5-fluorouracil

## Abstract

Resistance to chemotherapy is one of main obstacles in the treatment of colorectal cancer (CRC). However, the mechanisms are still unclear, and the treatment options are still limited. miR-543 has been indicated to act as an oncogene in some cancers, but its function in regulating chemoresistance has not been considered in CRC cells. This study investigated whether the down-regulation of miR-543 expression enhanced 5-fluorouracil (5-FU)-induced apoptosis in HCT8/FU colon cancer cells. In our study, qRT-PCR revealed that miR-543 expression was up-regulated in the HCT8/FU colon cancer cell line compared with that of HCT8 colon cancer cell line. An miR-543 inhibitor or mimic was transfected, followed by MTT assay to detect 5-FU sensitivity in HCT8 and HCT8/FU cell lines, which showed that IC_50_ of 5-FU was positively correlated with miR-543 expression. Further studies showed that miR-543 enhanced drug resistance by down-regulating the expression of phosphatase and tensin homolog (PTEN), which negatively regulates protein kinase B (AKT) activation. Additionally, an elevated expression of PTEN reversed the chemoresistance of miR-543-overexpressing HCT8 cells to 5-FU. These results indicate that miR-543 might be a target to increase the sensitivity of CRC cells to 5-FU through the PTEN/PI3K/AKT pathway.

## Introduction

Colorectal cancer (CRC) is the 4th most commonly diagnosed cancer (6.1% of the total cases) and the second leading cause of cancer-related mortality (9.2% of the total cancer deaths) in the world [1]. The 5-Fluorouracil (5-FU) has been used in the treatment of CRC for more than 50 years. In particular, the combination of 5-FU and leucovorin or methotrexate can improve the quality of life and survival in patients with advanced CRC [2,3]. However, many colorectal patients could not benefit from 5-FU because of the appearance of chemoresistance. Although resistance mechanisms have been extensively studied for 5-FU, therapies to target resistance pathways have yet to be identified [4].

MiRNAs are a kind of endogenously expressed small noncoding RNA molecules that are 20–24 nucleotides in length and possess many critical regulatory functions in cells [5]. MiRNA expressions are observed in some human malignancies, such as non-small-cell lung cancer (NSCLC) [6], CRC [7], and osteosarcoma [8]. In addition, miRNAs can also regulate chemoresistance in some cancer cells [9–12]. Several studies have reported that miR-543 de-regulation may promote events linked to tumor angiogenesis, metastasis, and invasion through different mechanisms [13,14]. Our previous study showed that miR-543 acts as an oncomiR in CRC and that its overexpression promotes migration and invasion in CRC cells [15]. However, the role of miR-543 in regulating chemoresistance in CRC cells remains largely unknown.

Phosphatase and tensin homolog (PTEN) is a tumor suppressor, and the loss of PTEN causing the formation of cancer has been confirmed [16,17]. Our previous study showed that PTEN can be regulated directly by miR-543 [15]. In the present study, we discovered that the down-regulation of miR-543 expression reduced the drug resistance of CRC cells to 5-FU by targeting PTEN.

## Materials and methods

### Cell culture

The HCT8 colon cancer cell line and HCT8/FU colon cancer cell line (5-FU-resistant) were purchased from MeiXuan Biological Science and Technology Ltd. (Shanghai, China). The HCT8 and HCT8/FU cells were cultured in RPMI-1640 medium (Bioind, Israel) supplemented with 10% FBS (HyClone, Logan, UT, U.S.A.), 100 mg/ml of streptomycin and 100 IU/ml of penicillin at 37°C under 5% CO_2_. HCT8/FU cells were incubated from HCT8 cells with increasing concentration of 5-FU until they could grow in medium with 5-FU (15 μg/ml) as normal HCT8 cells.

### Real-time PCR analysis

According to the manufacturer’s protocol, total RNA was extracted from homogenized cell samples with TRIzol reagent (Takara Bio, Otsu, Japan). For each sample, 6 μg of RNA from cell lines was used for reverse transcription with MMLV reverse transcriptase (Genepharma, Suzhou, China). The primer sequences were as follows: miR-543 forward: 5′- CAGTGCTAAAACATTCGCGG -3′ and reverse: 5′- TATGGTTGTTCACGACTCCTTCAC -3′; and U6 snRNA forward: 5′- CGCTTCGGCAGCACATATAC-3′, and reverse: 5′- TTCACGAATTTGCGTGTCATC-3′. Each PCR was conducted at 95˚C for 3 min, followed by 45 cycles at 95°C for 12 s and 62°C for 50 s. The expression of miR-543 was determined using Light Cycler 2.0 with the Light Cycler kit (Takara, Japan), and the U6 gene was used as the internal control for miR-543.

### Cell transfection and co-transfection

Transfection of the miR-543 mimic, the miR-543 mimic negative control (NC), the miR-543 inhibitor and the miR-543 inhibitor negative control (inNC) (Genepharma, Shanghai, China) was performed according to the manufacture’s instructions using Lipofectamine 3000 reagent (Invitrogen). PTEN (Myc-DDK-tagged)-human plasmid (Origene, U.S.A.) with an miR-543 mimic or pCMV6 (PTEN NC) with an miR-543 mimic were cotransfected into cell using Lipofectamine 3000 and p3000 (Invitrogen) according to the manufacturer’s protocol. Transfection efficiency was determined by qRT-PCR or Western blot assay in all experiments 24 h after transfection.

### Cell migration assay

The migratory capacity of the colon cancer cells was evaluated through a 24-well transwell plate. HCT8, HCT8/FU and HCT8 cells (miR-543 mimic tansfected or NC tansfected) cultivated in 6-well plates were digested with trypsin, 1.5 × 10^5^ cell in 300 μl of serum-free medium were plated in the upper chamber, and 650 μl of medium supplemented with 20% FBS was added to the lower chambers. After 24 h of incubation at 37°C, the cells that did not migrate were removed from the upper chambers. The cells at the bottom of the upper chamber that migrated were fixed with cold formaldehyde and stained with 0.1% crystal violet. The number of cells was counted in five random fields per chamber under a microscope at ×20 magnification.

### Cell proliferation analysis

For cell proliferation assays, cells in 6-well plates 24 h after transfection were plated to four 96-well plates (3000 per well). The results were collected at 24, 48, 72, and 96 h after plating. A 96-well plate was used each time. After two analyses, the medium was changed to the remaining two plates. For analysis, 4 h before the end of incubation, 10 μl of MTT (Sigma, U.S.A.) solution (5 mg/ml) was added to every well, and the plates were incubated for 4 h at 37°C. Then, 150 μl of DMSO (Sigma, U.S.A.) was added to each well after the supernatant was removed. Optical density (OD) values were measured at a wavelength of 490 nm in an ELISA 96-well microtiter plate reader (Bio-Rad 680, California, U.S.A.). All assays were performed in triplicate.

### IC50 and cell viability investigation

The HCT8 and HCT8/FU colon cancer cells were plated in 96-well plates at a density of 5000 cells/well. After 12 h, HCT8 and HCT8/FU cells were treated with 5-FU at different concentration for 48 h. The OD value at 490 nm were read on a Microplate Reader (Bio-Rad 680, California, U.S.A.) after treatment with MTT and DMSO. The IC_50_ value of 5-FU for each cell line was calculated using GraphPad Prism software (San Diego, CA, U.S.A.). Cell viability was calculated as the ratio of the OD value of the sample with 5-FU added to the OD value of the control sample.

### Western blot assays

Cell protein was extracted using RIPA buffer (Beyotime, Shanghai, China). After protein quantification, the equal weight protein lysates were separated by SDS gels, followed by the blotting on PVDF membranes (Millipore, Bedford, MA, U.S.A.). Then, the membranes were blocked with nonfat milk for 1.5 h and incubated with primary PTEN (Proteintech), phosphorylated protein kinase B (p-AKT, Abcam), protein kinase B (AKT, Proteintech), B-cell lymphoma 2 (Bcl-2, Proteintech), Bcl-2-associated protein X (BAX, Proteintech), p53 (Proteintech), p21 (Proteintech) or GAPDH (Proteintech) antibodies overnight. The next day, the membranes were incubated with horseradish peroxidase-conjugated secondary antibodies (Santa Cruz, CA, U.S.A.), followed by visualization using the ECL detection kit (Thermo scientific, Rockford, IL, U.S.A.). All assays were performed in triplicate.

### Flow cytology

To detect the cell apoptotic rate, cells were treated with the Annexin V-FITC apoptosis detection kit (Dojindo, Japan) according to the manufacturer’s protocol. HCT8 or HCT8/FU cells were seeded in 6-well plates that have been transfected for 12 h, and then 5-FU was applied at the IC_50_ value to cultured cells for 24 h. After digesting and washing with cold PBS three-times, the cells were resuspended in binding buffer solution, and the cell suspension density was approximately 10^6^/ml. A 100 μl cell suspension was stained with 5 μl of Annexin V-FITC (10 mg/ml) and 5 μl of propidium iodide (50 mg/ml) in the dark for 15 min at room temperature. Finally, the cell apoptotic rate was measured by flow cytometry (BD Biosciences, San Jose, CA, U.S.A.).

### Statistical analysis

All statistical analyses were performed using Graphpad Prism 5.0 and SPSS 17.0 (Chicago, IL, U.S.A.). Student’s t-test was used to analyze differences between groups. All data were presented as mean ± SD. When the *P*-value <0.05, the difference was considered to be statistically significant.

## Results

### MiR-543 can promote CRC cell migration but not proliferation

**Figure 1:**
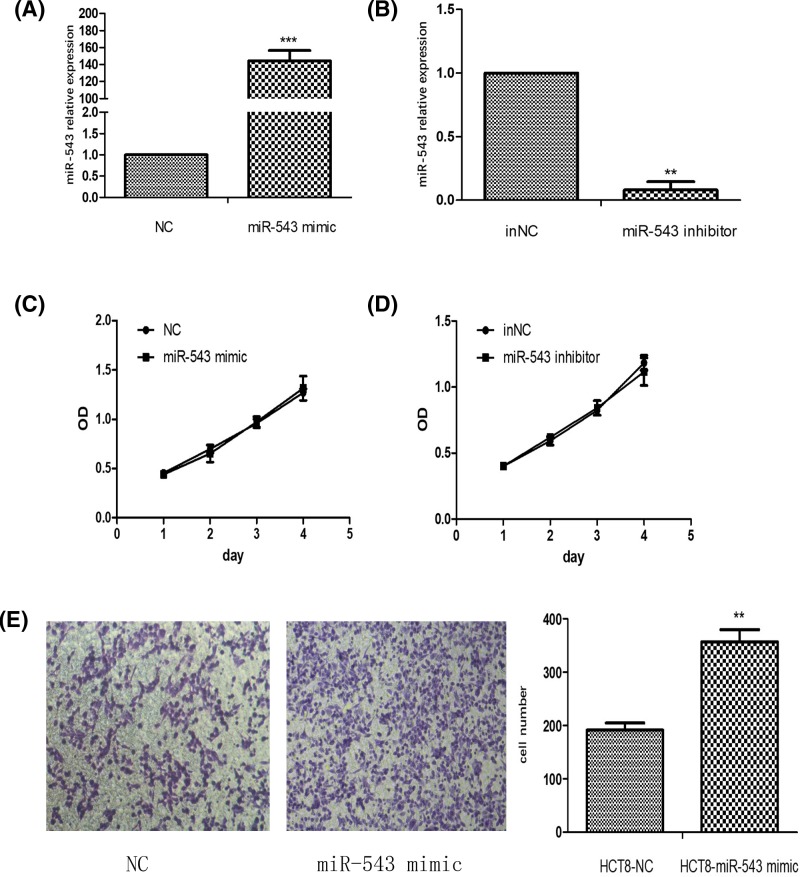
MiR-543 expression promoted CRC cell migration but not proliferation (**A**) The expression level of miR-543 in HCT8 cells transfected with the miR-543 mimic was measured by qRT-PCR analysis. ^***^P<0.001 vs NC. (**B**) The expression level of miR-543 in HCT8 cell transfected with the miR-543 inhibitor was measured by qRT-PCR analysis ^**^P<0.01 vs inNC. (**C**) Growth curves of HCT8 cells transfected with NC or the miR-543 mimic. (**D**) Growth curves of HCT8 cells transfected with inNC or the miR-543 inhibitor. (**E**) Cell migration was evaluated by a Transwell assay. Representative fields of invading cells on the membrane were observed by an inverted microscope (magnification, 20) **P<0.01 vs NC.

The miR-543 mimic and miR-543 inhibitor were transfected into the HCT8 cell line separately to validate the effect of miR-543 on CRC cell proliferation. The transfection efficiency was detected by qRT-PCR (Figure 1A,B). We examined that the overexpression of miR-543 has no influence on cell proliferation in HCT8 colon cell lines (Figure 1C). The miR-543 inhibitor also could not change the HCT8 cell proliferation rate (Figure 1D). We identified an elevated expression of miR-543 promoting HCT8 cell migration (Figure 1E), although we confirmed that miR-543 promotes CRC cell migration in HCT116 and SW480 CRC cell lines in our previous study [15].

### MiR-543 is up-regulated in HCT8/FU cells compared with HCT8 cells

**Figure 2:**
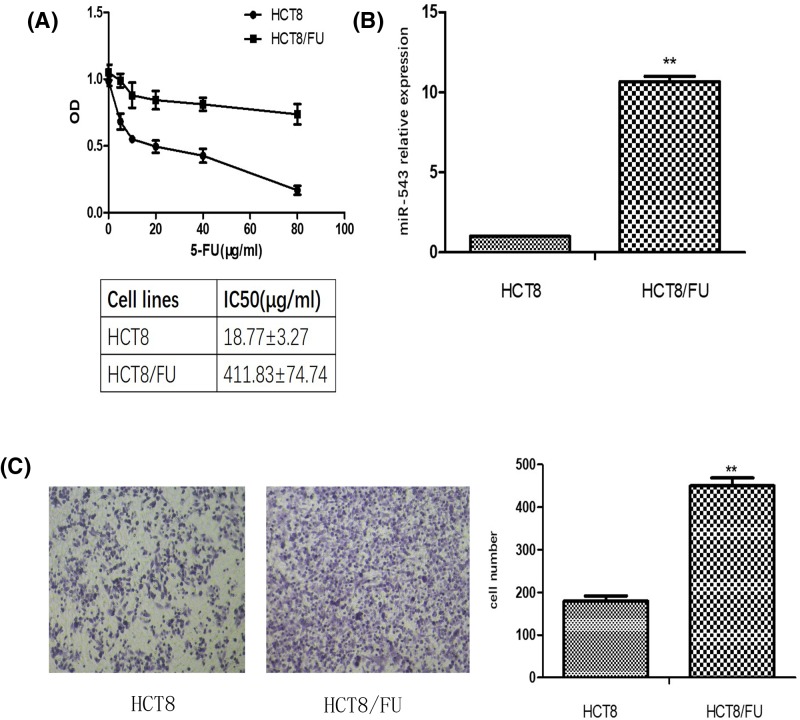
MiR-543 is up-regulated in HCT8/FU cells compared with its isogenic parental cells HCT8 (**A**) Dose–response curves of HCT8 and HCT8/FU cell lines towards 5-FU. The IC_50_ values are listed in the tables below. (**B**) The qRT-PCR analysis of miR-543 expression in HCT8 and HCT8/FU cell lines. ^**^P<0.01 vs HCT8; data represent the mean ± S.D.; *n*=3. (**C**) HCT8 and HCT8/FU cell migration ability were evaluated by a Transwell assay. Representative fields of invading cells on the membrane were observed by an inverted microscope (magnification, ×20) ^**^P<0.01 vs HCT8 cells.

Cell viability investigation showed that the growth of HCT8 and HCT8/FU cells treated with different concentrations of 5-FU after 48 h occurred in a dose-dependent manner (Figure 2A). To explore the potential relationship between miR-543 and the sensitivity of CRC to 5-FU, qRT-PCR was used, and the results showed that miR-543 is up-regulated in HCT8/FU cells compared with HCT8 cells (Figure 2B). Therefore, we chose HCT8 cells, which has a low level of miR-543 for up-regulation, and HCT8/FU cells, which has a high level of miR-543 for down-regulation. We further found that the migration ability of HCT8/FU cells was stronger than that of HCT8 cells (Figure 2C).

### Inhibition of miR-543 induces apoptosis in HCT8/FU cell line

**Figure 3:**
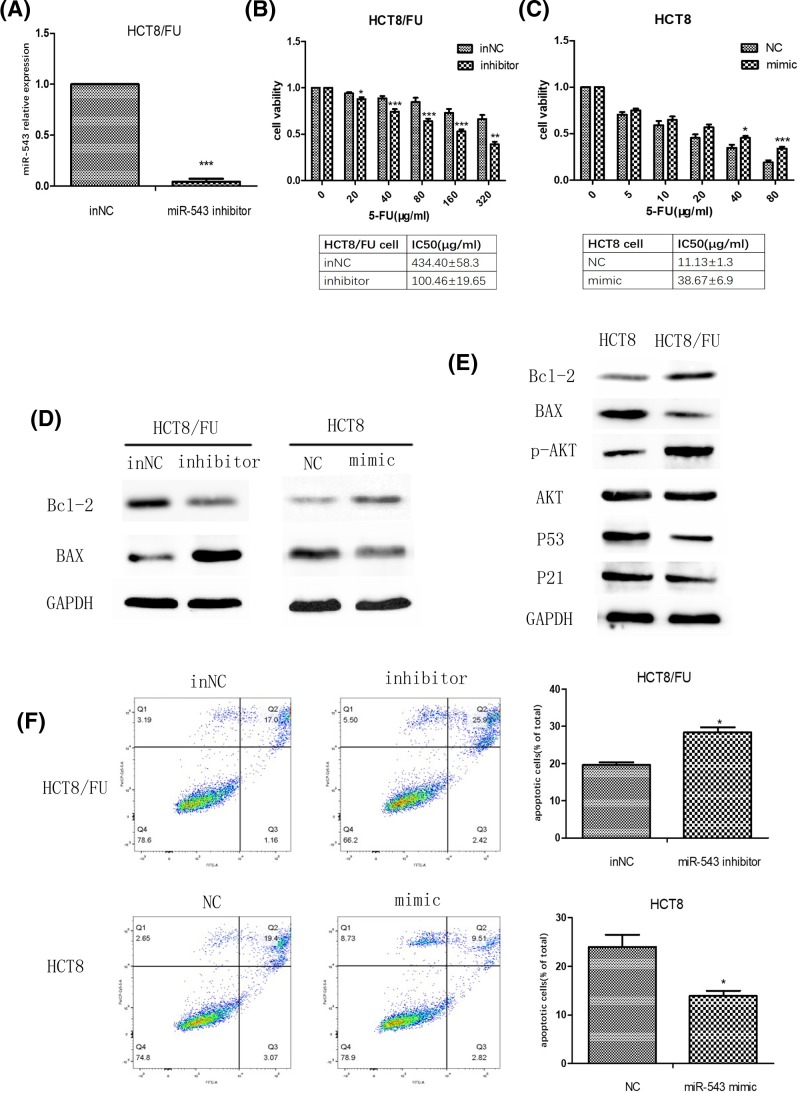
Effect of miR-543 expression on the chemosensitivity of CRC cells to 5-FU (**A**) Relative level of miR-543 in HCT8/FU cells transfected with the miR-543 inhibitor or inNC. (**B**) Dose–response curves of HCT8/FU cells transfected with the miR-543 inhibitor and its control towards 5-FU. IC_50_ values were listed in the tables below. (**C**) Dose–response curves of HCT8 cells transfected with the miR-543 mimic and its control towards 5-FU. IC_50_ values are listed in the tables below. (**D**). Protein expression levels of Bcl-2 and BAX in miR543-inhibitor-transfected HCT8/FU cells and in miR543-mimic-transfected HCT8 cells. (**E**) Protein expression levels of Bcl-2 and BAX in HCT8 cells and HCT8/FU cells. (**F**) HCT8/FU cells transfected with the miR-543 inhibitor and inNC and HCT8 cells transfected with the miR-543 mimic and inNC were treated with 5-FU for 24 h, followed by analysis of apoptosis. ^*^P<0.05 vs control; ^**^P<0.01 vs control; ^***^P<0.001 vs control. The data are presented as the mean ± S.D. of triplicate experiments.

To evaluate the effect of miR-543 on the chemoresistance of CRC cell, the miR-543 inhibitor and inNC were transfected into HCT8/FU cells. Transfection of HCT8/FU with the miR-543 inhibitor reduced miR-543 expression levels (Figure 3A). In addition, we found that the down-regulation of miR-543 expression weakens the resistance of HCT8/FU to 5-FU after a 48-h treatment, and the IC_50_ of the cell-transfected miR-543 inhibitor was 4-fold lower compared with the cell-transfected inNC (Figure 3B). Western blot results showed that transfection with the miR-543 inhibitor significantly down-regulated the expression of Bcl-2 (antiapoptosis protein) and elevated BAX protein levels in HCT8/FU cells (Figure 3D). Correspondingly, in HCT8 cells transfected with the miR-543 mimic, we observed the opposite results (Figure 3C,D). We found that Bcl-2 and p-AKT are highly expressed and that BAX, p53, and p21 are expression at low levels in HCT8/FU cells compared with HCT8 cells (Figure 3E). We also found that the protein expression level of total AKT did not differ between HCT8 cells and HCT8/FU cells (Figure 3E). Then, we detected the apoptosis rate of CRC cell induced by 5-FU by flow cytometry and found that the miR-543 inhibitor increased the HCT8/FU cell apoptosis rate and that the miR-543 mimic reduced HCT8 cells apoptosis rate (Figure 3F).

### MiR-543 promote the expression of PTEN in HCT8 and HCT8/FU cell lines

**Figure 4:**
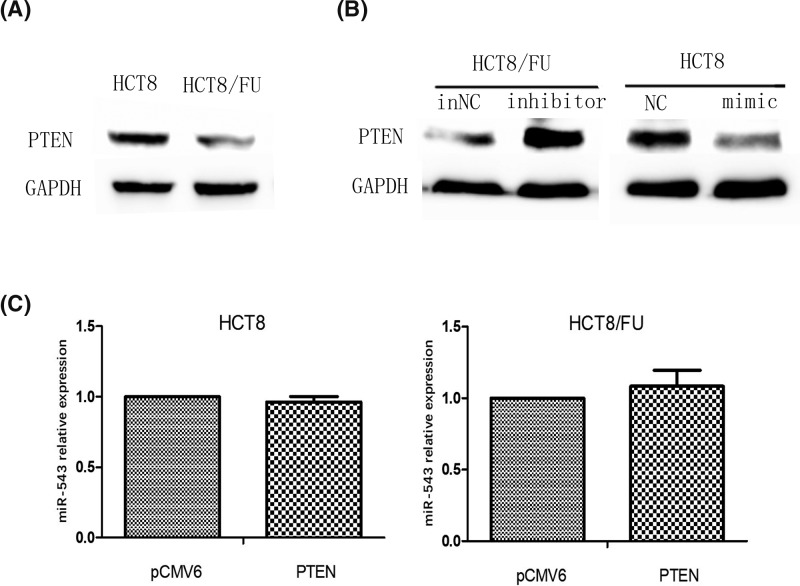
Alteration of miR-543 expression changed PTEN protein expression (**A**) Protein expression levels of PTEN were assayed by Western blotting in HCT8 and HCT8/FU cells. (**B**) Protein expression levels of PTEN in miR543-inhibitor-transfected HCT8/FU cells and miR543-mimic-transfected HCT8 cells. (**C**) The qRT-PCR analysis of miR-543 expression in HCT8 and HCT8/FU cell lines transfected with PTEN and pCMV6 (NC).

Our previous study showed that PTEN is a direct target of miR-543 through bioinformatic analysis and a dual-luciferase reporter assay [15]. To investigate the expression of PTEN in HCT8/FU, which has a high level of miR-543, a Western blot assay was employed. We found that PTEN proteins had a down-regulation trend in HCT8/FU cells compared with HCT8 cells (Figure 4A). Western blot also showed PTEN was down-regulated in HCT8 cell transfected with the miR-543 mimics; whereas, PTEN was up-regulated in HCT8/FU cell transfected with the miR-543 inhibitor (Figure 4B). Using qRT-PCR, we found that there was no significant difference in miR-543 expression between PTEN-overexpressing cells and their control (Figure 4C).

### PTEN reversed the effects of miR-543 on colon cancer cell chemoresistance

**Figure 5:**
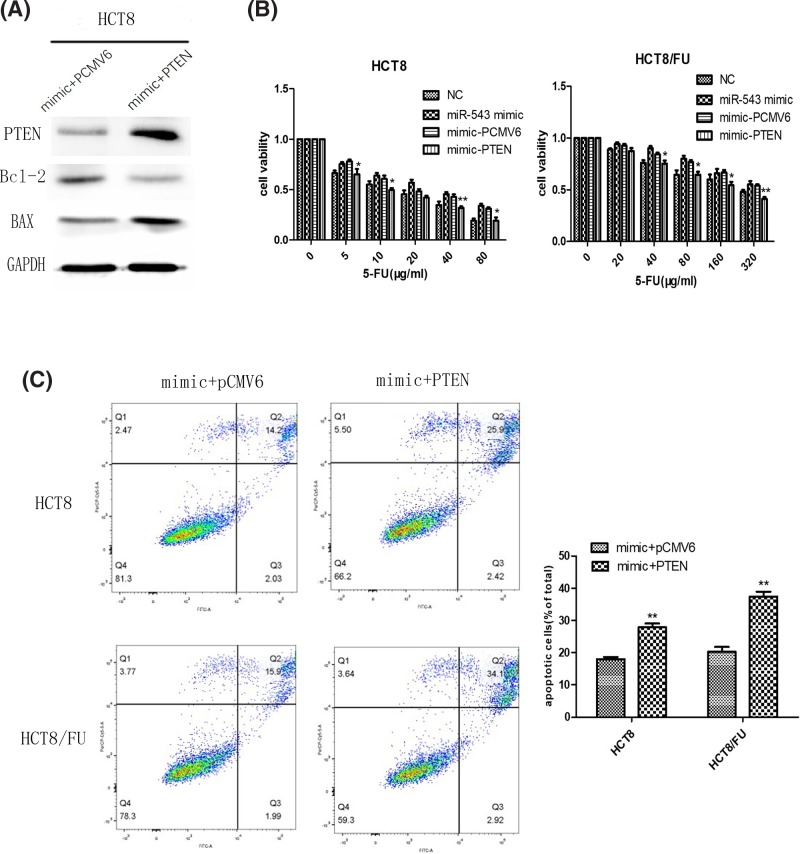
PTEN reversed the effects of miR-543 on colon cancer cell chemoresistance (**A**) Western blot analysis showed PTEN overexpression reversed miR-543 effect to the expression of Bcl-2 and BAX in HCT8 cell through the cotransfection of miR-543 mimic and PTEN. (**B**) HCT8 and HCT8/FU cells were treated with increasing concentrations of 5-FU for 48 h. MTT assays showed that overexpression PTEN re-sensitized miR-543-expressing cells to 5-FU treatment. The data are presented as the mean ± S.D. of triplicate experiments. ^*^P<0.05 vs mimic + pCMV6, ^**^P<0.01 vs mimic + pCMV6. (**C**) HCT8 and HCT8/FU transfected with miR-543 were transiently transfected with PTEN expression construct or pcmv6 (control vector). Up-regulation of PTEN increased cell sensitivity to 5-FU. ^**^P<0.01 vs mimic + PCMV6. The data are presented as the mean ± S.D. of triplicate experiments.

To consider whether the impact of miR-543 on CRC cell chemosensitivity was mediated by the inhibition of PTEN, we cotransfected the miR-543 mimic and PTEN plasmid into HCT8 cells. PTEN transfection efficiency was evaluated by Western blot analysis (Figure 5A). The overexpression of PTEN enhanced sensitivity to 5-FU in cotransfected cells compared with cells cotransfected with the miR-543 mimic and PCMV6, which suggests that PTEN reverses the effects of miR-543 (Figure 5B). Cell apoptosis assays indicated that the overexpression of PTEN markedly rescued the miR-543-induced enhancement of chemoresistance (Figure 5C). Western blot analysis showed that PTEN may reverse the expression of Bcl-2 and BAX in the HCT8 transfected miR-543 mimic (Figure 5A).

### MiR-543 inhibited the PTEN/PI3K/AKT pathway and activated apoptosis-related proteins

**Figure 6:**
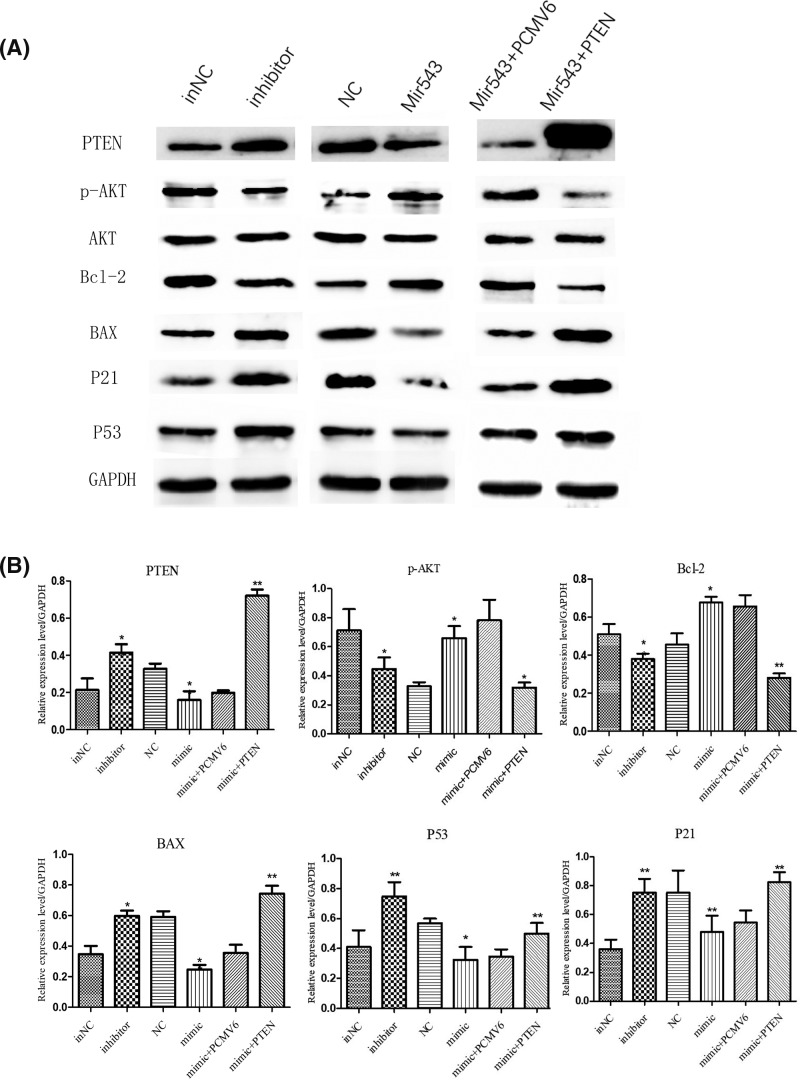
MiR-543 inhibited the PTEN/PI3K/AKT pathway and activated apoptosis-related proteins (**A**) Protein expression levels of PTEN, p-AKT, AKT, Bcl-2, BAX, P21, and P53 were assayed by Western blotting in HCT8 cells. (**B**) Statistical analysis. ^*^P<0.05 vs control; ^**^P<0.01 vs control. The data are presented as the mean ± S.D. of triplicate experiments.

We found that the miR-543 inhibitor up-regulate the expression of PTEN, BAX, p21, and p53 and down-regulated the expression of p-AKT and Bcl-2 in HCT8 cells (Figure 6). Furthermore, the overexpression of miR-543 down-regulated the expression of PTEN, p21, and p53 while simultaneously up-regulating the expression of p-AKT instead of total AKT (Figure 6). We cotransfected the miR-543 mimic and PTEN plasmid into HCT8 cells to investigate whether the overexpression of PTEN can reverse the effect of miR-543 on p-AKT. Western blotting showed that miR-543-PTEN attenuated the expression of phosphorylated AKT compared with miR-543-PCMV6; whereas, the total AKT level remained unchanged. In addition, p21 and p53 protein expression levels were elevated after PTEN was up-regulated (Figure 6).

## Discussion

MiRNAs play a critical role in different pathologies, ranging from metabolic diseases to cancer. Previous studies have reported that miRNAs have an impact on multidrug resistance and may be therapeutic targets in the clinic. For example, Zhang et al. [18] found that miR-587 inhibited the apoptosis induced by 5-FU and produced drug resistance in CRC. Hua et al. [19] showed that the overexpression of miR-1 increased chemosensitivity of NSCLC cells by inhibiting autophagy-related 3-mediated autophagy. Zhang reported that the inhibition of miR-425-5p increased the apoptosis induced by antitumor drugs by regulating PDCD10 in CRC cells [20]. Mir-543 acted as an oncogene to promote the invasion and migration of CRC [15], hepatocellular carcinoma [21], and gastric cancer [22]. In addition, it has been reported that in gefitinib-resistant NSCLC cell, miR-543 was up-regulated and promoted cell proliferation and invasion by targeting PTEN [23]. However, the link between miR-543 and drug resistance has not been reported in CRC, and we were the first to demonstrate that miR-543 promotes drug resistance in CRC treatment with 5-FU.

CRC is a high-risk malignant tumor in the digestive system worldwide; for decades following its discovery, 5-FU as a monotherapy has been moderately effective for improving the 12-month survival in CRC patients [24]. However, with the emergence of high rates of drug resistance, the 5-FU has not been the best choice in anticancer drugs for CRC therapy, even though it is combined with other chemotherapeutic agents. In the current study, we found that miR-543 is up-regulated in the HCT8/FU cell line and that the down-regulation of miR-543 increased the sensitivity of HCT8/FU to 5-FU. In contrast, the overexpression of miR-543 in HCT8 cells elevated its IC_50_ to 5-FU. Cell line evidence suggests that miR-543 may play a role in drug resistance in CRC. Western blot results showed that the protein level of miR-543 is directly proportional to Bcl-2 expression and inversely proportional to BAX expression *in vitro*. Bcl-2 is an antiapoptotic protein that can regulate the activation of the cellular apoptotic pathway and overexpression in many cancer [25–27]. For example, in diffuse large B-cell lymphoma (DLBCL) and chronic lymphocytic leukemia (CLL), Bcl-2 is important for cancer cell survival by limiting IP_3_R activity and regulating IP_3_ signaling [27]. BAX is a proapoptotic protein in the Bcl-2 family that is related to an increased apoptotic rate and leads to a better recovery in patients [25]. The destruction of the balance between Bcl-2 and BAX can result in tumor cells that are resistant to chemotherapy drugs [28]. Our study also found a positive correlation between miR-543 and Bcl-2 and a negative correlation between miR-543 and BAX.

PTEN is a kind of tumor suppressor gene that has powerful functions and is closely related to apoptosis [29]. Our previous studies have shown that PTEN is a direct target of miR-543 through bioinformatic analysis and a dual-luciferase reporter assay [15]. In the current study, we showed that overexpression of miR-543 can down-regulate the expression of PTEN and that an miR-543 inhibitor can elevate PTEN expression in CRC cells. The loss of PTEN can activate the PI3K/AKT signaling pathway, which is responsible for carcinogenesis, progression, and metastasis [30–32]. Dave et al. [33] reported that breast cancer cells with PIK3CA mutations were resistant to trastuzumab when the expression of PTEN was down-regulated. It is worth noting that AKT is able to elevate Bcl-2 level through the PI3K/AKT/Bcl-2 axis [34,35]. Moreover, Matsunaga et al [36] found leukemic cell acquired resistance to anoikis or drug-induced apoptosis via the PI3K/AKT/Bcl-2 signaling pathway. We showed that the down-regulation of miR-543 resulted in a significant decrease in the level of AKT phosphorylation and that the up-regulation of miR-543 induced a high expression of p-AKT. The total AKT has never changed in the two conditions. The overexpression of PTEN to HCT8 cells transfected with the miR-543 mimic simultaneously reversed the p-AKT level and increased apoptosis rate of 5-FU in colon cell lines.

Tumor cell apoptosis is often associated with reactive oxygen species (ROS) and tumor suppressor p53 [37,38]. Particularly, p53 and its downstream targets CDK-inhibitor p21 play a critical role in tumor cell proliferation and apoptosis [39]. In this study, we observed that the protein levels of p53 and p21 were regulated by miR-543, of which the change was inconsistent with p-AKT in the p53^wt^ colon carcinoma cell line HCT8. Recent studies have reported that regulating the AKT/p53 signaling pathway can inhibit CRC cell and MCF-7 breast cancer cell growth and metastasis [40,41]. Li [42] et al. also showed that crocetin and cisplatin induced esophageal cancer cell apoptosis by up-regulating the p53/p21 pathway. Considering above results, we strongly suggest that miR-543 may represent a new therapeutic target for overcoming chemoresistance in CRC.

**Figure 7:**
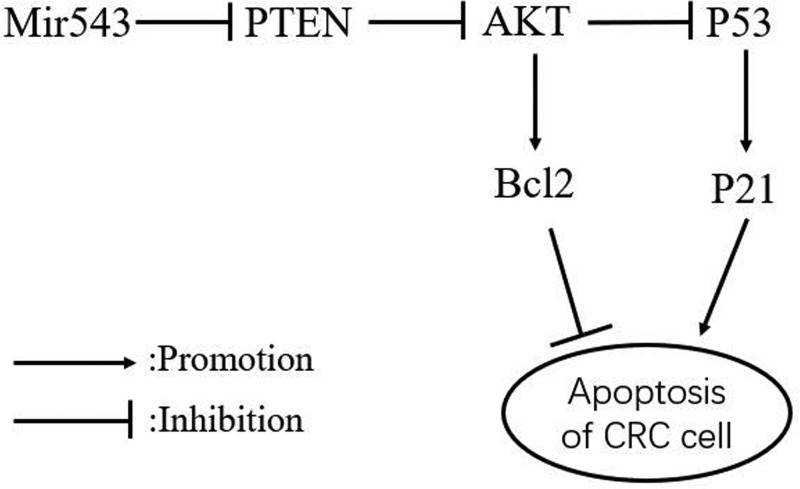
Proposed model of MiR-543 attenuate sensitivity of colon cancer cells to 5FU-induced apoptosis

In conclusion, we have elucidated that miR-543 enhances the resistance of CRC cells to 5-FU and that the down-regulation of miR-543 increases the sensitivity of CRC cell to 5-FU through suppressing PTEN/PI3K/AKT signaling pathway, as shown in Figure 7. The results of this study provide new insights for the development of drugs that inhibit the expression of miR-543 to enhance the efficacy of 5-FU in CRC patients. Considering that the experiments were only carried out *in vitro*, the next step can be explored at the animal level.

## Abbreviations

5-FU: 5-fluorouracil
AKT: protein kinase B
BAX: Bcl-2-associated protein X
Bcl-2: B-cell lymphoma 2
CRC: colorectal cancer
GAPDH: glyceraldehyde-3-phosphata dehydrogenase
inNC: inhibitor negative control
NC: negative control
NSCLC: non-small-cell lung cancer
OD: optical density
P-AKT: phosphorylated-protein kinase B
PDCD: programmed cell death
PI3K: phosphatidylinositol 3-kinase
PTEN: phosphatase and tensin homolog
qRT-PCR: quantitative real time polymerase chain reaction
RIPA: radio immunoprecipitation assay
