# Correction: Down-regulation of miR-543 expression increases the sensitivity of colorectal cancer cells to 5-Fluorouracil through the PTEN/PI3K/AKT pathway

**DOI:** 10.1042/BSR-2019-0249_COR

**Published:** 2022-01-18

**Keywords:** 5-fluorouracil, colorectal cancer, MicroRNA-543, PTEN

The authors of the original article “Down-regulation of miR-543 expression increases the sensitivity of colorectal cancer cells to 5-Fluorouracil through the PTEN/PI3K/AKT pathway” (*Biosci Rep* (2019) **39**(3), https://doi.org/10.1042/BSR20190249) would like to correct Figure 3, as they had placed the wrong image within panel F. The authors confirm that this Correction does not alter the conclusions of their original study.

**Figure 3:**
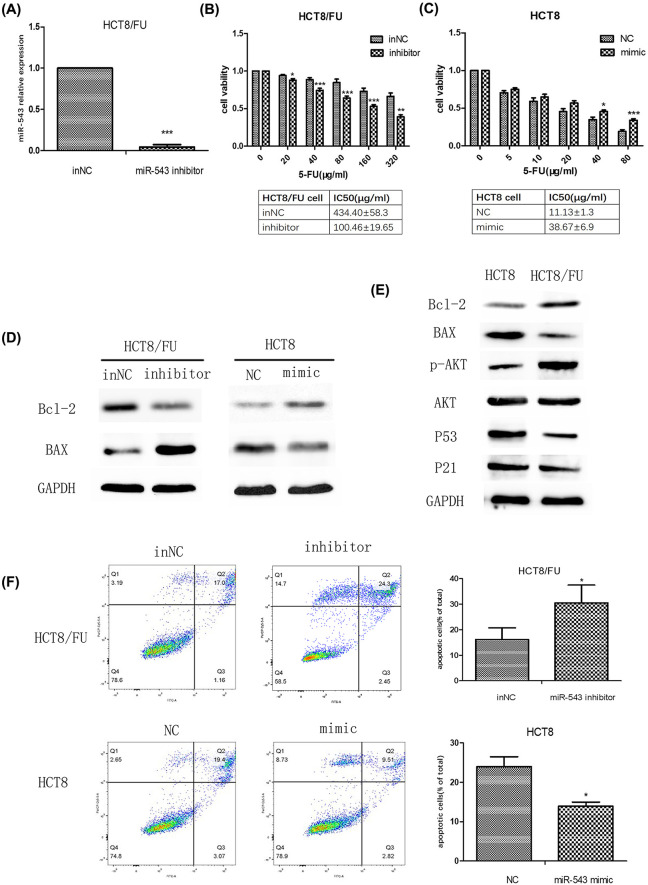
Effect of miR-543 expression on the chemosensitivity of CRC cells to 5-FU (**A**) Relative level of miR-543 in HCT8/FU cells transfected with the miR-543 inhibitor or inNC. (**B**) Dose–response curves of HCT8/FU cells transfected with the miR-543 inhibitor and its control towards 5-FU. IC_50_ values were listed in the tables below. (**C**) Dose–response curves of HCT8 cells transfected with the miR-543 mimic and its control towards 5-FU. IC_50_ values are listed in the tables below. (**D**). Protein expression levels of Bcl-2 and BAX in miR543-inhibitor-transfected HCT8/FU cells and in miR543-mimic-transfected HCT8 cells. (**E**) Protein expression levels of Bcl-2 and BAX in HCT8 cells and HCT8/FU cells. (**F**) HCT8/FU cells transfected with the miR-543 inhibitor and inNC and HCT8 cells transfected with the miR-543 mimic and inNC were treated with 5-FU for 24 h, followed by analysis of apoptosis. *P<0.05 vs control; **P<0.01 vs control; ***P<0.001 vs control. The data are presented as the mean ± S.D. of triplicate experiments.

